# Community and Healthcare Providers' Perspectives on Male Circumcision: A Multi-Centric Qualitative Study in India

**DOI:** 10.1371/journal.pone.0091213

**Published:** 2014-03-10

**Authors:** Seema Sahay, Karikalan Nagarajan, Sanjay Mehendale, Sibnath Deb, Abhilasha Gupta, Shalini Bharat, Shripad Bhatt, Athokpam Bijesh Kumar, Vidisha Kanthe, Anju Sinha, Nomita Chandhiok

**Affiliations:** 1 National AIDS Research Institute, Pune, India; 2 National Institute of Epidemiology, Chennai, India; 3 Pondicherry University, Puducherry, India; 4 HRRC, LLRM College, Meerut, India; 5 Tata Institute of Social Sciences, Pune, India; 6 RMRC, Belgaum, India; 7 Indian Council of Medical Research, Delhi, India; Edinburgh University, United Kingdom

## Abstract

**Background:**

Although male circumcision (MC) is recommended as an HIV prevention option, the religious, cultural and biomedical dimensions of its feasibility, acceptability and practice in India have not been explored till date. This study explores beliefs, experiences and understanding of the community and healthcare providers (HCPs) about adult MC as an HIV prevention option in India.

**Methods:**

This qualitative study covered 134 in-depth interviews from Belgaum, Kolkata, Meerut and Mumbai cities of India. Of these, 62 respondents were the members of circumcising (CC)/non-circumcising communities (NCC); including medically and traditionally circumcised men, parents of circumcised children, spouses of circumcised men, and religious clerics. Additionally, 58 registered healthcare providers (RHCPs) such as general and pediatric surgeons, pediatricians, skin and venereal disease specialists, general practitioners, and operation theatre nurses were interviewed. Fourteen traditional circumcisers were also interviewed. The data were coded and analyzed in QSR NUD*IST ver. 6.0. The study has not explored the participants' views about neonatal versus adult circumcision.

**Results:**

Members of CC/NCC, traditional circumcisers and RCHPs expressed sharp religious sensitivities around the issue of MC. Six themes emerged: Male circumcision as the religious rite; Multiple meanings of MC: MC for ‘religious identity/privilege/sacrifice’ or ‘hygiene’; MC inflicts pain and cost; Medical indications outweigh faith; Hesitation exists in accepting ‘foreign’ evidence supporting MC; and communication is the key for acceptance of MCs. Medical indications could make members of NCC accept MC following appropriate counseling. Majority of the RHCPs demanded local in-country evidence.

**Conclusion:**

HCPs must educate high-risk groups regarding the preventive and therapeutic role of MC. Communities need to discuss and create new social norms about male circumcision for better societal acceptance especially among the NCC. Feasibility studies on MC as an individual specific option for the high risk groups in health care setting needs to be explored.

## Introduction

The World Health Organization (WHO) has estimated that globally 30% of males aged 15 and over are circumcised, with almost 70% of these being Muslim [1]. Evidence from the past observational and meta-analytical studies and clinical trials on male circumcision (MC) has demonstrated its protective role in reducing the HIV/STI transmission by nearly 50-60% [2], [3], [4], [5], [6]. MC is since being advocated as a potential HIV prevention method in developing countries with high burden of HIV. Wide scale acceptance of MC is expected to save huge monetary investment in other HIV prevention programs [7]. With a burden of 2.09 million people living with HIV/AIDS and an estimated 1.16 million new infections, HIV remains a public health problem in India [8]. It has also been shown that between 2000 and 2011, the male and female sex ratio of new HIV infections has remained close to 40% - 60% in favor of males in India. However, increasing HIV prevalence along with increasing new HIV infection rates in the previously low prevalence states of north India [8] are an issue of concern for the health program managers.

In view of the continuing challenge of the HIV disease burden in India and the WHO recommendation to add MC to the existing national HIV prevention programs [9], it is vitally important to understand the meaning and practice of MC in a multi-religious and multi-ethnic country like India. Any biomedical prevention technology is effective only if it is accepted by the community. Hence acceptability of male circumcision as an HIV prevention option needs to be studied to gain insights into the local cultural and environmental context of use of this option. India has approximately 120 million circumcising populations living [10] together with 900 million non circumcising populations (NCC). Circumcision in India is inherently linked to minority religions of Islam and Judaism practiced by Muslims and Jewish people respectively. The majority population belonging to the Hindu religion represents the non circumcising community in India. The NCC considers circumcision as a religious practice among Muslims and hence hard to relate with. This is very unlike the African scenario where circumcision is widely practiced even among non-Islamic populations [10], [11]. Religious sensitivity in India coupled with factors like illiteracy and conservativeness is likely to pose major challenges in promotion and acceptance of MC, similar to the opposition faced by the family planning program in India in the past [12]. Apprehensions for surgery and pain have also been reported to pose additional barriers for promoting and accepting male circumcision at many places [2], [13], [14], [15], [16].

As the idea of circumcision as a public health intervention is gaining support; multiple social, anthropological and ethnographic studies on circumcision have been conducted in various African countries in the past decade to understand history, symbolism, related socio-cultural concepts, and philosophical principles associated with MC [17]. There is a dearth of similar studies in India with the exception of the following three: 1) a study in Pune reported biological protection due to MC among circumcised men attending STD clinics but did not report on its acceptability [18]; 2) a small scale regional study conducted in Mysore city of south India explored the acceptance of male circumcision for HIV prevention among mothers belonging to the NCC [19]; 3) a study in Kerala state in south India assessed the willingness of healthcare practitioners to provide male circumcision services to STI clinic attendees [20]. The latter two studies, both from south India, have reported general acceptance to MC among the study participants. However, these studies have the limitation of lack of generalizability of the findings to the diverse Indian populations due to the unrepresentative nature of their samples. Consequently, there is very little known about the religious and cultural dimensions of MC practice and also about its biomedical aspects. A critical gap in knowledge is about perceptions and practice of MC among the communities. India has an estimated 2.1 million people living with HIV/AIDS (PLHA) with an estimated prevalence of 0.32% and 0.22% among sexually active males and females respectively in 2011 [21]. Hence in spite of foreseeable cultural barriers, acceptance of a scientifically proven intervention such as MC needs to be explored. On this background a multisite task force study of Indian Council of Medical Research (ICMR) was planned. This study aimed at understanding beliefs, experiences, meaning and perceptions of MC practice and prevalent medical needs, practices and understanding about MC as an HIV prevention option among stakeholders such as various community representatives as well as healthcare providers. This large scale qualitative study, the first of its kind pertaining to male circumcision practice, is likely to provide guidance in making recommendations to the national policy and program regarding introduction of male circumcision as a bio-medical intervention for HIV/AIDS prevention in India.

## Methodology

### Ethics statement

The study was approved by the institutional ethics committees of National AIDS Research Institute (Pune) vide letter dated 18 March, Tata Institute of Social Sciences (Mumbai) vide letter dated 23 August, 2008, LLRM College, (Meerut), RMRC (Belgaum) vide letter no. RMRC/ICMR/EC/218/07-08, dated 20 Feb. 2008 and Calcutta University (Kolkata) vide letter dated 18 February, 2008. Written informed consents were obtained from the study participants.

### Study sites and context

The study was carried out between June 2009 and June 2011 among the members of circumcising and non-circumcising communities (CC& NCC respectively) and healthcare providers using qualitative research method. We conducted a series of in-depth interviews and key informant interviews among the respondents selected from four cities in four states in India viz. Meerut (Uttar Pradesh state, Northern India), Kolkata (West Bengal state, Eastern India), Belgaum (Karnataka state, Southern India) and Mumbai (Maharashtra state, Western India). These sites were conveniently selected from respective geographic regions of India.

### Sampling and data collection

Uniform sampling and data collection methods were employed across all the four study sites. The study included a community component (CC & NCC) and a healthcare providers' component at all the study sites. Purposive sampling technique was used to enroll 134 participants representing both the study components. The recorded interviews were transcribed verbatim, translated in English language and typed in electronic format at the study sites and were sent within 2-3 days to the coordinating site. At the coordinating site, two researchers and the Principle Investigator read interviews and if required, the sites were instructed to conduct repeat interviews of the respondents to obtain missing information or to substantiate some information using the same routine of data collection and processing. Generally repeat information started emerging after 3 interviews in almost all the categories and if data saturation did not happen even after 5 interviews, the study sites were instructed to conduct more interviews or repeat some of the interviews.

In the community category, 62 in-depth interviews were conducted among members of CC and NCC represented by medically and traditionally circumcised men, parents of circumcised children, spouses of circumcised men, and clergy from different religions. In the healthcare providers' category, 58 registered healthcare providers (RHCPs) including general and pediatric surgeons, pediatricians, STD and general practitioners (allopath/homeopath/ayurveda/unani practitioners) and operation theatre (OT) nurses were interviewed. Fourteen traditional circumcisers were also interviewed.

#### Recruitment

Teams from two coordinating institutes (Indian Council of Medical Research (ICMR), New Delhi & National AIDS Research Institute (NARI), Pune), and four collaborating institutes (Tata Institute of Social Sciences (TISS), Mumbai; Regional Medical Research Centre (RMRC), Belgaum; Department of Psychology, University of Calcutta, Kolkata & Human Reproductive Research Centre (HRRC), LLRM College, Meerut) conducted the study. Identifying and locating the participants was a challenge in case of medically circumcised men, parents of recently circumcised children and traditional circumcisers. The participant recruiters and/or interviewers with Masters level social science background received uniform training to work with the communities by establishing liaison with non-governmental organizations (NGOs) and community gatekeepers for recruitment of study respondents. The study teams received training in qualitative research methods, human subject research issues and communication skills.

For both the study components, participants were identified and recruited through personal contacts and referrals from NGOs and religious clerics or other stakeholders. Consenting community respondents from the non-circumcising community, mainly the medically circumcised men, parents of recently circumcised children and spouses of circumcised men were approached through the doctors who had performed circumcisions. The religious clerics were approached through the members of their respective religions who volunteered to facilitate the contact. Members of circumcising community were approached through local contacts in the CC community or through a recruiter belonging to the CC community within the research team or the institute. Snow-ball approach was also utilized to get new respondents for the study.

After receiving the contact details of a respondent, the study interviewer fixed appointment with the respondent briefly informing him/her about the study and requested for a quiet and confidential location for the interview. Before the interview, a written informed consent was obtained from the respondent and interview was conducted at the location chosen by the respondent. Most of the HCPs and traditional circumcisers preferred the location of their own work set up while others preferred their homes or NGO office. The interviews were audio recorded after obtaining permission for recording from the respondents.

The interviews were conducted in respective local languages at the sites (*Bengali, Kannada, Marathi, and Hindi*) or in English.

### Study tools

Separate in-depth interview guidelines using probes were used for RHCPs and the community respectively. The open ended questions allowed interviewees to answer in their own words and offer their personal experiences [22]. This type of interviewing strategy gave interviewers sufficient flexibility to probe areas of interest and to gain a deeper understanding of the issues discussed. Through head nods or verbal cues, study participants were encouraged to talk at length about their experiences.

The focus of the interview for the community was on awareness regarding prevalent MC practice in India, attitude, perception/belief, religious practices, opinions on medical male circumcision and family planning methods among men, MC among non circumcising community, willingness to accept MC after understanding that MC is an efficacious HIV prevention option for men, messages needed to promote MC and barriers and facilitators of MC as an HIV prevention option. The focus of the interview for RHCPs and traditional circumcisers was on knowledge about MC and the actual procedure, medical conditions for MC, difference between traditional and medical male circumcision, opinion about MC as an HIV prevention option, attitude and beliefs about MC, infrastructure required for MC, opinion on appropriate age of MC, advocacy needs for MC, barriers and facilitators, willingness for MC, their role in promoting MC, training received, need for training others as circumcisers and patients' concerns when MC was advised. During the interviews some additional probing was done on traditional circumcisers.

The guidelines were translated into relevant local languages. The guidelines were pilot tested in 2 RHCPs and 4 community members in the study areas. In India, MC is a religious practice and the communities can be arbitrarily divided into CCs and NCCs wherein CCs [mostly Muslims] practice traditional male circumcision. Thus most of experiential knowledge about traditional MC is derived from CCs and anecdotal knowledge is derived from NCCs. Also, experiential knowledge about medical MC is derived from NCCs. We have explored the existing practices of traditional circumcision among Muslim male children and adult male circumcision due to medical reasons as well as the future acceptability for MC. The study has not explored the participants' views about neonatal versus adult circumcision. The study aimed at getting deeper understanding of the influence of traditional and religious MC upon the potential acceptability of adult MC in the country.

### Confidentiality

In view of the religious sensitivity around the issue of MC in India, we employed a broader focus of male controlled biological HIV prevention options instead of male circumcision alone for our study. They were explained about the sensitivity of the subject of inquiry and given an option to store their signed copy of the informed consent form (ICF) at the site rather than with them. However, none of the participants refused to take the copy of the ICF with them. Respondents were given unique numeric IDs and data forms or audio recordings were linked with them but not with their names. Names of the study participants were recorded only on the ICF which were stored separately [and not with the data files], under lock and key which was accessible only to one authorized person at each site. Final anonymized data was stored at the coordinating site but the informed consent forms remained in the custody of site investigators.

### Data analysis

The data derived from the interviews typically formed the largest corpus of unstructured textual material. The transcripts were read and re-read over and over again by the three researchers [ABK, SS and VK] at National AIDS Research Institute (NARI), the coordinating site and coded by in NUD*IST(version 6.0) software using a thematic approach [23]. These themes were discussed with the entire research team in a workshop mode. The initial broad themes were developed on the basis of interview guide but new codes and themes emerged from the data. Component specific thematic patterns were triangulated for both the study components of HCPs and community. The key points extracted from the text were assigned a series of ‘codes’. Quotes and analytical memos were reviewed by two authors (SS, KN) to identify common themes and variant views. As an iterative process, excerpts were re-read and the initial broad codes were further detailed into sub-themes using a grounded theory approach to developing fine codes [24] The definitions of these emerging fine codes were then discussed and refined within the research team and reports were generated for further readings and analysis. Illustrative quotations that most clearly represent each of the identified theme were chosen to be included in the manuscript [25]. Overall, 51 repeat interviews were conducted (Mumbai – 1; Belgaum – 8; Kolkata – 25; Meerut – 17) either to confirm some findings or to attain data saturation. In this paper, verbatim excerpts from the interviews are presented as translated English versions of their original narration in *Hindi*, *Bengali*, *Kannad*a, or *Marathi*. Vernacular phrases or terms are italicized with an explanation in brackets. The square brackets in the verbatim excerpt indicate explanation/meaning provided by the research team.

## Results

A total of 189 potential respondents were approached of which 134 respondents participated in this study. They included 62 CC/NCC representatives, 58 RHCPs and 14 traditional circumcisers [Table 1].

**Table 1:** A brief overview of study participants describing the number of study participants by their geographical location, professional background, community background and gender.

Characteristics	Sub-categories	No. of study participants N = 134
**Geographic area of India (n = 134)**	**Region (n = 134)**	
	North – Meerut	32
	West – Mumbai	34
	South – Belgaum	35
	East - Kolkata	33
**Health Care Providers (n = 90)**	**Type of Health Care Providers (n = 90)**	
	Registered health care providers	58
	Traditional Circumcisers	14
	Religious Leaders	18
**Community (n = 44)**	**Context of MC practice (n = 44/44)**	
	Circumcising community	27
	Non-circumcising community	17 [7 were medically circumcised]
	**Religious leader (n = 18/44)**	
	Hindu	5
	Muslim/Islam	9
	Jewish	1
	Others	3
	**Gender (n = 44/44)**	
	Women	18
	Men	26

Of the 55 refusals to participate in the study 38, 2, 7 and 8 were from Belgaum, Meerut, Mumbai and Kolkata respectively for reasons such as paucity of time or unwillingness to talk about MC which was practiced only among certain religions.

Irrespective of the religious background, age, profession and geographical location; every single participant of the study was aware of a cultural/religious practice called male circumcision. Most of the members of the NCC identified MC as a practice followed predominantly by the Muslim community (people practicing Islam religion) in India. None of the NCC members were aware that Jewish people (practicing Judaism) in India also practiced MC. All Muslims, with the exception of one woman, knew that MC is not a religious practice of other communities.

Using the NUD*IST software, 47 nodes and sub nodes emerged related to knowledge and source of knowledge of contraception, knowledge of male circumcision, perceptions about MC, age of circumcision, reason and experiences, circumcisers, brand image, cultural practice, acceptability, complications, service gaps, decision making, religious identity, sensitization, sensitivity, profile, requirements, HIV knowledge and MC as HIV prevention option. Repeated readings, memo writing, and discussion on cases and excerpts by the research team led to the following six themes: 1) Male circumcision: the religious rite, 2) Multiple meanings: ‘religious identity/privilege/sacrifice’ and ‘hygiene’, 3) Circumcision entails pain, surgery and cost, 4) Medical indications outweigh faith,5) Hesitation exists in accepting ‘foreign’ evidence supporting MC and 6) Communication is the key for acceptance of MC.

### Male Circumcision: the religious rite

Traditional male circumcision, termed as *“khatna”* or *“sunnat”*, was reported to be traditionally performed by circumcisers of the Muslim community or barbers who were denoted by various names like z*arrah, khalifa, mullah, nai, hajjam* and *nau*. Although traditional circumcision was not performed by any particular caste among the CCs, one traditional circumciser from Meerut informed that a separate sect called as *“salmani biradari”* or *“sheikh biradari”* was meant to perform MC. The RHCPs noted that very few members of the CC, usually from the high socio-economic strata and higher educational background opted for circumcision in hospitals at the hands of doctors. This was confirmed by respondents representing CC. They also reported that traditional circumcision was performed among affluent families as a celebration and in poor families as a small ritual sometimes without any ceremony. Presence of *moulvi* (an Islamic religious cleric) was not considered necessary for MC and the preferred places to perform the procedure were either the house or the backyard of a mosque. A mother from CC from Mumbai said (23 yrs) (code-1-2-034), “*It*'*s [/male circumcision/]) like a wedding. All the rites performed at the time of wedding, are done [/performed during MC/]. And during the circumcision process “Bismillah” [/prayer/] is read and the “poophi” [/paternal aunt/] would stand on one leg with “Quran e Shareef” [/Islamic religious book/] on her head*”. A mother representing CC from Belgaum (35 yrs) (code-2-2-085) explained the practice of burying the cut penile foreskin during circumcision: *“The thing is that, the skin [/foreskin/] that is large [/long/], it is pulled, and how much it is large [/depending on how long it is/], it has to be cut. After cutting they fold that skin and bury it”.* A Jewish traditional circumciser from Mumbai (60 yrs) (code-1-1-019) also mentioned that burying the cut foreskin is an important ritual.

Many ‘*Hindus*’ (essentially representing NCC who follow Hindu religion in India) compared male circumcision rituals among Muslims with their own rituals such as *munjui* (purification of body in childhood), *onnoprashon* (first rice eating ceremony of child), *jasoothan, naamkaran* (naming ceremony), and *poite* (sacred thread ceremony).

### Multiple meanings: ‘Religious identity/privilege/sacrifice’ and ‘hygiene’

A strong religious commitment was expressed by the circumcising community towards MC, which was reported to be a mandatory ritual for all male members with no exception allowed. Various beliefs such as *“it*'*s a vow to god”, “it*'*s a stamp to Islam”, “it is our right to undergo”, “it*'*s a rule and order to do”, “you become Muslim after circumcision”, “it is the command of Allah [/God/]” and “it is the desire of Allah that his follower be clean”*. existed among the CC.

Irrespective of the caste differences within the circumcising community like *Saifies, Salmanis, Ansaries, and Maliks*, circumcision was practiced universally and the man who had undergone circumcision was called “*sunnate Ibrahimi*” and “*sunnate Mohamadi*”. The Hindus considered MC as “*musalmani operation*”. More than a religious commitment, circumcision was equated with religious identity [in this case ‘Muslim’] in India, which differentiated Muslims from non circumcising communities following other religions. An individual was said to be a Muslim from the ‘day’ he was circumcised and not from being born of Muslim parents. Being circumcised was considered to be an important sacrifice. A traditionally circumcised Muslim man (32 yrs) (code-1-2-026) from Mumbai commented, *“I mean to say that 100 martyrs are counted as equivalent to one circumcision. The benefits that lord bestows on 100 martyrs are also received by a circumcised [individual]”.* Although some of the CC members felt that it was their “*farz*” (duty as per Islamic faith) to undergo MC, there were others who felt that circumcision is actually not a “*farz*” and people practice male circumcision of their own free will because they have faith in this particular practice.

Jewish community in India was also reported to be practicing male circumcision. Respondents from this community also expressed a strong belief in male circumcision as their religious identity. MC in this community is traditionally performed on the eighth day of the birth of the male child and it has been described as “*each person*'*s contract” or “command of Abraham”*. Historical anecdotes were shared by the respondents to emphasize significance of MC. It was stated that circumcision was performed for Jewish children during World War II and even in concentration camps. A Jewish cleric from Mumbai (55 yrs) (code-1-2-032) said, *“Every Jew is duty-bound to get his son circumcised and it is a question of the child*'*s identity … as a boy is given a name only after this rite has been performed.”*

The Hindu clergymen identified the practice of male circumcision with the Muslim community. An NCC *pujari* [/a Hindu cleric/] from Kolkata in West Bengal, India, male (35 yrs) (code-3-2-133) said, *“Yes I do know that they [/Muslim community/] have a belief that if one is not circumcised, that person is not a Muslim. Like we Hindus inherit our religion by birth, I guess they don*'*t, otherwise this compulsory circumcision would not have cropped up [/practiced/]”.* The practice of male circumcision is a kind of ‘argument’ between religious sects about having faith or not having faith: *“With the circumcision done, and having read the kalma [/prayer/] the person [/child/] is accepted as a Muslim; without khatna [/circumcision/] he is considered a ‘Hindu’…this is what is there in this religion-* explained a 32 year old CC woman in Mumbai (code-1-2-029).

The RHCPs as a whole felt that CC opts for male circumcision solely for religious purposes. A Christian religious leader identified MC as essentially a Muslim practice, which was long before banned in *“Baptism”* for religious reasons in the Christian community.

In this study, out of the 14 traditional circumcisers, although two mentioned that they performed circumcision for the purpose of hygiene rather than religion; they generally believed that the practice of MC was linked to religious faith as well as beliefs regarding its hygienic benefits. Societal norms of not allowing a non circumcised Muslim to marry or enter *“jammat”* (society) and offer prayers in mosques were the critical drivers for enduring faith in male circumcision in CC. It was also reported that a man belonging to the NCC having an intention to marry a woman from CC had to undergo MC. In one of the sites, all the traditional circumcisers interviewed (n = 4) informed that they had also done circumcision for religious conversion purposes among NCCs.

Some CC members articulated beliefs not related to religion but hygiene supporting the practice of MC. It was mentioned that urine spillage on the worn garments makes them feel unhygienic and unfit for offering prayers. The practice of MC or “*khatna*” helps to avoid spillage of urine. The link between hygiene and ‘Islam’ was an integral part of their religious faith. The word “*nisful-imann*” means that hygiene is a critical component of Islam religion. The intention behind the practice of removal of the foreskin of penis in MC is to get rid of the “*gande bacche”* (smegma/collection under the foreskin) thereby making the person “*gandgi* free” (free from dirt or filth). The CC also believed that MC helps in prevention of certain diseases. However, many NCC respondents questioned beliefs of CC about MC and hygiene: “*…But for hygiene? I*'*m sorry but I won*'*t allow it [/accept it/]. In India, 60% of people are non-circumcised, so [does] that mean [/that/] they don*'*t maintain hygienic habits? I*'*ll prefer teaching my child the necessary hygienic habits than make him undergo circumcision.”*-Voice of a married 45 year old woman from NCC in Kolkata (code-3-2-126).

### Circumcision entails pain, surgery and cost

Out of the total 27 CC members interviewed, one traditionally circumcised man was totally opposed to MC due to pain experienced during the procedure, and two married women from CC were not willing to accept the practice of MC for their male kin. Pain was an experience that was not easily forgotten by both the CC and NCC members who had undergone the procedure. Parents of NCC whose children were required to be circumcised, protecting them from pain was important. A 29 year old female general practitioner from NCC in Kolkata (code-3-1-103) mentioned: “*Because whatever information you gave, says that it is* [/*MC*/] *not vaccine against the disease … nor does it give any guarantee that HIV would never be there if you are circumcised. So, for just say 75% chance … why should I allow my son to go through the pain? Easier would be to wear a condom. If it* [/*MC*/] *acted as a vaccine I would have definitely gone for it*.”

Members of CC undergoing MC also shared their experience of pain that they had not forgotten even in adulthood: “I used to be just eight [when I underwent MC]; it is painful. So even now, when I think about the time [/when I underwent MC/] first that painful [/first thing that I recollect is that it was painful/] but the other thing is... I had to stay in the house and then I couldn't urinate... and just quite, it's not a nice experience”- Voice of a 55 year old cleric belonging to CC from Mumbai (code-1-2-032). Another traditionally circumcised 35 year old man from Mumbai (code-1-2-039), while talking about pros and cons of traditional circumcision recalled, “Now it's better [when people can access male circumcision services at the hospitals], see, at that time [/earlier when there was no facility in the hospitals/], it was different. I remember my father had got that razor which he got sterilized and that put some local anesthesia and there was some doctor uncle of mine who was there[/He means that during those days when anesthesia was not used for MC, his father had asked a doctor to be present/]. But ultimately it's a painful experience [/Despite anesthesia and sharp razor used during his circumcision in the presence of a ‘doctor’ friend also, pain was an unforgettable experience/]”. However, owing to the traditional importance of MC, many felt that the pain can be endured for such an important rite and hymns are recited to reduce pain as shared by a 24 year old married woman (code-1-2-038) belonging to CC in Mumbai, “…is only important to pray by reciting ‘Rakat Shukarana’ [/to pay tributes to God/], such that the child suffers less because of this recitation”. Another 47 year old Christian male cleric (code-4-2-185) from Meerut confirmed this viewpoint, “Now-a-days they celebrate it as ceremony, so that the child becomes happy and does not think about pain. He only thinks about the ceremony”.

Some of the members of CC opined that in the present time circumcision is considered more of a ritual and practiced not due to faith alone but for its social acceptance. Like Muslims (CC), the reasons given by Jewish people (CC) were also based on religion and health beliefs around MC. A Jewish cleric from Mumbai (55 yrs) (code-1-2-032) mentioned: *“Male Circumcision is definitely not only religious but all laws based on the Jewish religion are formed on pure health reasons”.* Another viewpoint from NCC was that circumcision was practiced among CCs owing to the custom of multiple marriages (polygyny), risk of venereal diseases and practices like not taking bath.

NCC also expressed concern about the cost of surgery but religion invariably influenced the decision making process to get MC done even if it was prescribed by the doctor. A 54 years old male pediatric surgeon (code-1-1-003) belonging to NCC shared, *“Mostly the patients are scared of surgery. We try to convince them by saying this [/male circumcision/] is a small surgery and it will take less time to recover. But sometimes they try to look for lesser [/other non surgical and affordable/] options i.e.no surgery, no anesthesia, no staying at hospital, no expenses and most important they think that after surgery it [/penis/] will look like Muslim people.”*

Cost emerged as another issue if male circumcision is to be offered in programmatic mode for HIV prevention. Several RHCPs at all 4 sites opined that medical male circumcision might be costly: “*there has to be an operation theatre, anesthesia, ward boys, helper, necessary equipment, operation theatre time, nurse time and surgeon time. The approximate cost for the surgery would be [/INR/] 3000/- [approximately 50 USD]”-* An RHCP at Mumbai (code-1-1-002).

If male circumcision is positioned as ‘medical male circumcision’, the cost of MC would go up because it would be a surgery rather than a rite; latter being affordable to all. A man (code-3-2-131) belonging to CC talked about his fears about the cost of ‘surgery’ or medical male circumcision and underlying fear of stigma, *“Yes, that [medical male circumcision] partially fulfills my wishes. At least my son won*'*t suffer much. But you know what; I can*'*t afford the cost of the ‘operation’ [/medical male circumcision/]. There are some hajjams [/barbers/] who are good at heart and do it for free… I can always lie [in case I get medical male circumcision done which might not be so well accepted in his community i.e. CC] and then say that it has been done by a barber [/traditional circumciser/] of Kolkata [Hence I would satisfy my community]. But when I can*'*t afford the cost of the operation [/medical MC/], I can*'*t do anything to save my child from the pain”.*

Other CCs also raised similar issues. They commented that the cost of the operation and pain associated with the procedure might make MC less acceptable among NCC members even if offered as an HIV prevention option. A traditionally circumcised man from CC in Kolkata (27 yrs) (code-3-2-131) gave an important perspective regarding acceptability of medical male circumcision in the NCC in low socio-economic strata, *“No one wants to be operated. People are scared of operation and pain. And moreover poor people like me won*'*t be able to afford the operation and you can*'*t expect a ‘Hindu’ [/NCC/] to undergo circumcision through the hands of a Zarrah [/traditional circumciser which is an affordable option/], they won*'*t believe [/have faith/] in them [/skills of traditional circumcisers/].”*

### Medical indications outweigh faith

RHCPs provided an elaborate list of clinical conditions for which medical circumcision is recommended in children and adults. The common conditions cited were *phimosis and related conditions, urinary tract infection [UTI], warts in prepucial skin, vesico - rectal reflux in children, sexually transmitted diseases, diabetic infection, vitiligo of prepuce and pain during intercourse*. The overall experience of RHCPs indicated that the most important reason for NCC to undergo male circumcision, disregarding any religious or social barriers, was the severity of pain and urination related problems due to phimosis and urinary tract infections. A pediatric surgeon from Kolkata (male/NCC/60 yrs) (code-3-1-110) shared, *“…because they are in such a pain, they are willing to do anything and at that time faith or religion doesn*'*t come in the scene. A couple of days they just need to inform their respective families and talk about it. That*'*s all…”* Similarly another STD practitioner from Mumbai (male/Hindu/29 yrs) (code-1-1-004) pointed out that intensity of the medical problem trivializes most of the issues surrounding MC, *“The social barriers and religious taboos are overlooked by the people if they are advised surgery on medical grounds.*”

A change in attitude towards MC was observed among NCC members if someone in their families had undergone MC. A Hindu mother from an orthodox caste in Mumbai (31 yrs) (code-1-2-040) who reported that her two children were circumcised explained: *“But I guess more and more it [/MC/] is [*being*] done for hygiene purposes. Now I have heard that a lot of children got it done after my sons got it done probably because I started talking about it”.*

Out of 7 medically circumcised men from NCC community, everyone expressed urination problem as the main reason for getting circumcised. It was also noted that of all the NCCs who opted for or acknowledged the practice of circumcision, none had sought permission or discussed with their religious clerics about it and they relied greatly on the doctor's suggestions and had acted due to their own medical problems. One STD practitioner from Meerut (Male/NCC/30 yrs) (code-4-1-153) observed, *“They [/*people from NCC who are advised MC*/] quite agree. They have no problem if the problem [/medical problem/] gets relieved, by saying that it is ok [/to* agree for MC*/]! Doctor Sahib [/a salutation for doctor/] has told us regarding MC and we have to get it done”.* A general practitioner in Mumbai (CC/Male/50 yrs) (code-1-1-015) also shared the same experience, *“We have advised [MC] to some NCC people … those who are suffering from…. Because there is no other alternative treatment except [MC]… So most of the patients, you can say 80 to 85%, they follow [/the advice/]”*. Some of the respondents from the NCC simply annulled the prevalent association of male circumcision with CC religion. A medically circumcised man from NCC in Mumbai (36 yrs) (code-1-2-042) dismissed linking MC with Islam religion, “*That is called lack of knowledge … not in ours [/Hindu religion/], it [/lack of knowledge/] is in Muslim people [who believe that MC is for religious purpose], not in ours … means…nothing… nothing comes [/religion does not come in the way when we undergo male circumcision*/]. *My own circumcision has been done. For my two children also it has been done in childhood…”*

It was noticed that few women from NCC, who had opted for MC of their male children, considered MC as an “operation” done for medical reasons and tried to disregard its religious linkages. One mother from NCC from Mumbai (50 yrs) (code-1-2-033) said, “*If there is this [/medical/] problem … we do not call it khatna. We call it an operation”*.

Participants of both genders from the NCC did not report the feared social discrimination associated with male circumcision. Of all the 7 medically circumcised men from NCC, there was not a single man who reported experience of social stigma or out casting. A medically circumcised man from NCC from Meerut (30 yrs) (code-4-2-183) said, “*I have got it [/circumcision/] done and after this …. I want that my other friends also go for this because it is nice thing”.* A mother from NCC (Mumbai/31 yrs) (code-1-2-040) added, *“…I have friends ….they are also…Brahmins [/an NCC upper caste in India associated with religious rituals/] and she [/the friend/] was telling me that her entire family had to go through this [/MC/]. All the males in the family…for medical purpose…[had done MC].”*

A common phenomenon noted by RHCPs was that all of their patients from NCC had asked for alternatives initially, sought second opinion from other doctors and took from one week to few weeks to make the final decision to undergo circumcision. There were occasions, where some patients requested RHCPs for alternative surgical options, without the foreskin being removed. Some respondents from the CC mentioned that males from NCC who had undergone medical male circumcision, later on voluntarily opted for MC of their male children to avoid future medical complications that they themselves had experienced. In addition, there were reported instances when adults underwent circumcision, prior to getting married for gaining sexual pleasure.

### Hesitation exists in accepting ‘foreign’ evidence supporting MC

The information that MC is efficacious in preventing HIV acquisition among men brought mixed response of pride, happiness, mis-interpretations as well as caution and concern among the members of the CC. It was noted that among all the RHCPs, one private practitioner from Meerut (Male/Hindu/42 yrs) (code-4-1-157) plainly rejected MC on religious grounds. In contrast to the feelings noted among the CC members, most of the RHCPs and the NCCs, did not accept the information regarding efficacy of MC for HIV prevention. A 48 year old male STD practitioner (code-3-1-119) in Kolkata questioned the evidence. “*It [/MC/] does not/it cannot reduce the [HIV] transmission as such, though it can reduce the risk factor to a slight weight [/to some degree/], but it cannot eliminate, or cannot be relied upon, and cannot be suggested [/recommended/]…as a prevention option, and no data has shown that Muslim population is suffering from AIDS any less than Hindus”.* Another 48 year old male RHCP from NCC in Belgaum (code-4-1-157) in south India held a similar view, “*If we assume circumcision as a protector then we are wrong. It is completely wrong and it is not like this that if someone is circumcised then that person will not be infected by HIV. So, the Muslims and the Hindus may get infected from HIV even if they are circumcised”.* Another RHCP (code-4-1-165) from north India in Meerut mentioned, “*No, we don*'*t accept anything by one study. If a person says anything [/recommends/] on the account of one study it will not be valuable… this surgery [/circumcision/] has no role in prevention of HIV/STD.”* All these excerpts emerged even after the interviewer discussed the results of several efficacy studies with the study participants.

Majority of the RHCPs questioned the available scientific evidence supporting the role of circumcision in HIV prevention and felt that it was ‘foreign’ in nature as none of the trials were conducted in India. Out of all 58 RHCPs, 10 strongly rejected the idea of promoting MC as an HIV prevention option owing to the lack of scientific evidence in the Indian context. Rest of the RHCPs also had some reservations. Providing information on the benefits of circumcision based on available global evidence did not produce any significant change in their opinions about acceptance of MC. However, a surgeon from Mumbai (Male/Hindu/) (code-1-1-006) mentioned, *“I would say that if it is scientifically proved by large scale randomized controlled trials and it effectively shows that it is helpful in reducing the incidence and prevalence of HIV over a period of time ‘here’ [/in India/] only then it should be promoted”.* Almost all the RHCPs in this study who belonged either to CC or NCC, believed that promotion of MC in the NCC (especially Hindus) as a mass program will face mass resistance due to associated religious sentiments. If propagated as an HIV prevention strategy, MC may add to already prevailing stigma among communities about HIV. Behavioral dis-inhibition and false sense of security were the other perceived concerns following the suggestion of programmatic introduction of MC. An RHCP from Kolkata (code-3-1-121) mentioned, “*Now the illiterate men who are driven mainly by common sense and common information, they will think that when doctors are saying that you do circumcision, then they feel they will not get HIV.”*

Hindu, Sikh and Jain clerics representing NCCs (n = 9) expressed unwillingness to support MC. However, one Hindu cleric from Kolkata was reluctantly willing to accept the MC option on medical grounds. Many others gave warning alerts that any proposal to promote male circumcision would be unacceptable and would meet with extreme reactions from various religious sects in India. A 29 year old cleric from NCC at Meerut in north India (code-4-1-179) explained, *“…Result will be that Hindus will not accept this [/circumcision/], religious riots may take place”.* The reasons for rejection were purely based on religious grounds like *‘circumcision is not given [/written/recommended/] in NCC shastras [/scriptures/]”, “God*'*s creativity should not be disturbed”, and “purna shareer [/absolute body in its natural form/] should not be altered or modified”*. Tension between CCs and NCCs surfaced very clearly. A female nurse from NCC in Kolkata, (40 yrs) (code-3-1-112) said, *“If the removal of one structure  = name of a historical controversial religious structure =  can become a political issue in India then you are talking about something what mass thinks it to be [/considers/] the religious identity”! [/an expression of concerned shock on her face/]*.

Some participants opined about the gender norms prevalent in the country. “May be they [/people/communities/] wouldn't accept [MC] readily as it happened in case of vasectomy. Vasectomy has become a national program but still the requests [/acceptance by people/] for the same are very less than tubal ligation. It [/tubal ligation/] is a far more common practice. People won't readily accept because ‘the male’ has to undergo the procedure [/circumcision/]. They won't even get ready to use condom how they would undergo surgical procedure?”-Voice of a 44 year old pediatric surgeon (code-1-1-002) from NCC in Mumbai.

Unlike Hindu clerics (NCC), although Christian clerics were not supportive of circumcision as a regular practice on religious grounds, they were willing to accept it with some reservations due to its medical benefits. Some of the RHCPs talked of the possibility that MC might get accepted slowly like the family planning program that had initially experienced some resistance in the country but now the program is running smoothly. An STD practitioner from Mumbai (Male/Hindu/29 yrs) (code-1-1-004) stated, “*if there are benefits of circumcision then people will start accepting it on their own … but will start accepting 5, 10 may be 20 years [later]”.*

### Communication is the key for acceptance of MCs

The perceptions in the CC about the acceptance of male circumcision in the NCC were mixed. They were of the opinion that the NCC will categorically reject any proposal from the CC about benefits and efficacy of MC. For example, even among health care providers, resistance was observed. A 40 year old operation theatre nurse (code-1-1-005) belonging to the NCC in Mumbai said, *“I don*'*t think people especially Hindus will accept [MC]. When there are other available methods of prevention [against HIV] then why to opt for circumcision?”*

But among the 17 respondents representing community members belonging to the NCC, only one married woman from Kolkata (45 yrs) (code-3-2-126) categorically rejected circumcision even on medical grounds. However, she changed her opinion after the post-interview debriefing regarding the global evidence of role of MC in HIV prevention by the study staff. Appropriate advice from the doctors was deemed important. It appeared that illiterate and rural people from NCC are likely to show low levels of acceptance to MC and more efforts will have to be taken to educate the community.

Although the RHCPs did talk about religious reservations for acceptance of MC by their patients, there was not a single NCC respondent who was not willing to undergo MC if medically required. This underscores the need for appropriate communications regarding MC as an essential medical intervention for specific medical conditions or an HIV prevention option. Individuals or families of NCC who had an experience of circumcision were favorably inclined to accept the same in their families without reservations. A Hindu mother from Meerut (32 yrs) (code-4-2-184) explained, “*If my child/husband catches a disease [/AIDS/] which may become serious in future then, in my opinion he should get the circumcision done”* and she also added, “*I can tell my friends that look, my husband is circumcised and he did not experience any problem since he had got his circumcision done*”.

Of the 58 RHCPs, 12 (21%) (codes: 1-1-001; 1-1-015; 1-1-017; 2-1-055; 2-1-057; 2-1-065; 3-1-106; 3-1-111; 4-1-152; 4-1-154; 4-1-158; 4-1-167) belonging to CC (n = 4) and NCC (n = 8) strongly accepted MC as a prevention option for HIV. Most of the RHCPs felt that if the benefits, evidence and procedures of circumcision were conveyed to the community appropriately, acceptance could be better in the NCC. A general practitioner from Belgaum (CC/Male/70 yrs) (code-2-1-055) added *“As a doctor … certainly, I would recommend definitely to everybody…. now if you go to foreign countries … America, England, Germany … there, all the male children are circumcised”*. This comment also brings out prevalent misconceptions even among doctors about MC and the need to educate them as well.

Many respondents recommended that doctors, health workers or people from the NCC alone should be involved in promoting MC to gain acceptance by the NCC. They also said that before giving any kind of message, awareness must be created about male circumcision in all sections of the community.

The idea of making MC as one of the HIV preventive options in addition to condoms was strongly supported by all RHCPs and a few of them were also willing to advice it, but mainly to the high risk groups. Among the community members, a 28 year old medically circumcised man from NCC in Belgaum (code -2-2-077) said “*First we have to see towards limited persons [/specific high risk population/], we need to go through medical practitioners … we need to tell doctors. Doctors will consult [/counsel/] them [/persons practicing high risk behaviors/], those who are suffering from sexually transmitted diseases and those who are ‘near to get’ [/at higher risk of acquisition/] sufferings [/infection/] from sexually transmitted disease and those who have multi sexual partners, homosexual… these people need to consult doctors”*

A major focus and thrust on male circumcision is likely to face immense challenges in India. Besides religious issues, MC is likely to further aggravate HIV related stigma. A 60 year old male surgeon from NCC (code-3-1-111) clearly warns, “*This is where [/what/] you people don*'*t understand. Suppose everyone knows that circumcision reduces the risk of HIV/AIDS! The next person who gets circumcised would be looked upon as someone who is getting [/MC done/] for prevention of HIV/AIDS, but the poor fellow may be getting circumcised for urinary infections. You see, our society is heavily stigmatized by HIV/AIDS. You simply have to remove the stigma, before any campaigning for MC is done to remove [/prevent/] the disease [/HIV/]. Because until and unless you remove the stigma, people won*'*t come forward, they are scared of the society that they will be identified as HIV positive people.”*

## Discussion

The custom of circumcision as a religious rite is prevalent among Muslims at all the four study sites located in the eastern, western, northern and southern parts of India and also among Jewish people studied in western part. MC in India is accepted as a traditional practice of CC commonly performed by traditional circumcisers among male children. Child male circumcision among CCs is a traditional practice following parental and family assent. World over, the issue of consent process required for religious, traditional and non-therapeutic child circumcision is debatable and unresolved [26], [27], [28], [29].

To adhere to the custom of male circumcision practiced by their communities, few affluent and empowered CC members might opt for circumcision of their male child at the hands of qualified doctors instead of traditional circumcisers. Thus in India, it is important to differentiate between MC as a traditional practice and MC done for medical reasons. Traditional circumcision can be performed either by a clinician or by a traditional circumciser with implicit consent of parent and it is only practiced among CCs whereas medical circumcision is the ready option for the NCCs whenever medically indicated.

This study has explored the views on male circumcision as a potential HIV prevention option in India. Religion emerged as a major determinant of acceptability of circumcision in India as MC is universally associated with Islam religion and could have major challenges in its acceptability among followers of other religions in the country [30], [31]. Traditional circumcision is an important rite de passage ceremony celebrated for neonates and infants and sometimes children up to 7-8 years. It is practiced and celebrated among both poor and rich classes in the circumcising community. Traditionally, circumcision is performed by traditional circumcisers often without an anesthetic as reported by several other authors [31], [32], [33], [34], [35].

Male circumcision is like a qualifying step for full membership in the religion and establishing an individual's position in the society in the CCs. It is also believed to improve genital hygiene and help prevent sexually transmitted diseases. Various religious faiths practicing circumcision believe that MC is a *“religious duty”* irrespective of the class, creed, caste, profession, and geographical location. This practice has also been reported as a ‘religious duty’ in other parts of the world [11], [36]. MC is considered as a “purification” rite (denoted by word “sunnat”) and it makes the concerned person fit for offering prayer to “Allah”, the God. Conceptually comparable purification rites are prevalent in other religions of NCC in India as well but they are not as universally practiced as MC among CCs in India.

It is important to understand various dimensions of the practice of MC in India from *emic* i.e. insider's perspective rather than *etic* perspective of an outsider; the latter is mainly concerned with clinical issues. Due to heavy influence of religion and religion based practice of MC, NCC seems to have conservative views regarding acceptance of MC. Traditional circumcision, which is practiced for very young children, might not be acceptable to the NCC. However, the NCC had the awareness regarding traditional MC as well as experience of medical MC. It is to be understood that even while discussing adult MC, the reference point for NCC or RHCP or any other Indian is the ‘male neonatal/child circumcision’ that represents the traditional circumcision practiced among CCs in India. In the traditional ‘male neonatal/child circumcision’ in India, consent has no bearing; parents belonging to CC accept and adhere to the practice in case of their male children; else they would not be accepted as members of the community. The rite is often associated with a public ceremony. Consent of parents is considered implicit. Since, public health service/program pertaining to male circumcision are not implemented in India; the only experience is that of traditional childhood circumcision or medical MC among adults for treatment purposes. The willingness or concerns of adults for allowing their male children or kin to undergo MC can be considered as proxy indicators of their acceptance of MC. This does not necessarily indicate acceptance or concerns for ‘neonatal’ circumcision as this is beyond the scope of this study. It appears to be too early for India to dissociate MC from its label of religious practice among CCs. Consent for medical male circumcision is a standard procedure pertaining to any surgery in health facility.

The study reveals challenges in positioning adult MC as an HIV prevention option in the Indian society. The acceptability of MC in India seems to be dependent on religion, stigma, and cultural disposition and information provided.

### Religion as cohesive force in circumcising and non circumcising communities in India

Male circumcision is a religious faith based ritual which results in cohesion and bonding within the practicing community. However, it also results in polarization with opposing views between CC and NCC. French sociologist Émile Durkheim claims that religion attempts to offer a singular answer to life [37] to sustain cohesion and solidarity between its practitioners. To sustain faith in MC, Muslims have largely relied on the traditional circumcision and simplification of this procedure. MC is performed by the traditional circumcisers who are easily accessible and affordable to the community. It can be performed anywhere, by anyone and without the intervention of any cleric, thus removing any religious officialdom and procedural complexity, yet solidarity is very much apparent within the followers of the Islamic religion. Significance lies in the association of MC with public ceremony or festivities which probably reflects visible support to the religious practice and ensures adherence to the custom. The corollary is that the NCC in India shows solidarity and cohesion in the opposite direction. Medicalization of circumcision has improved access to MC in the Western milieu. Although scholars have been desensitized towards it, the split between ritualistic versus medical circumcision continues to occur [38].

The historical occurrence of communal tensions and riots between the CC and NCC in India wherein circumcision was used as a mark to ascertain religious identity, and ban on circumcision issued by religious fundamental activists of the NCC adds much to the complexity linked with male circumcision in India, when compared to other countries [12], [39]. Thus despite the obvious promise of male circumcision as an HIV prevention tool, advocates of the procedure face a wide range of challenges worldwide [40].

### Need for circumventing stigma and cost

Although medically advised MC was found to be acceptable in the study settings, there was an associated stigma and fear of discrimination. The NCC was more comfortable to consider medically indicated circumcisions as ‘operations’. RHCPs too recounted the decision making process following the advice for MC in the NCC and requests from people for providing other alternatives instead of MC. It is important to be sensitive to cultural norms of decision-making process in case of a culturally contentious issue. This process is useful because it is always accompanied with cognitive restructuring in favor of the existing custom. It was observed that those who were inclined to opt for circumcision were also prepared to disregard the pain and trauma associated with the procedure of MC and also tended to exaggerate its potential health benefits. The acceptance of MC in the NCC could very well be explained using Health Belief Model (HBM) [41], in which various psychological and social factors are believed to influence an individual's decision regarding a beneficial health action [42]. The severity of phimosis and UTI related conditions drives the NCC to undergo MC without considering any social and religious taboos. Viewed from the constructs of the HBM, the results of this study show, that while “religious belief” was acting as a *“modifying factor”*, *“doctors”* were playing a crucial part in making *“cues of action”* rather than *“religious clerics”*, which led the individuals from the NCC to undergo MC. The differentiation of MC as an *“operation”* rather than *“khatna”* by the mothers from the NCC, reveals the underlying HBM dynamics in this study, where the importance of *“perceived medical benefits”* had overpowered the *“perceived religious barriers”*, which is similar to the findings of a quantitative study conducted in Botswana, in which the “experience of circumcision” emerged as strong predictor for undergoing MC [13].

The individuals from the NCC who had undergone male circumcision often tended to deny its religious connotations and reported acceptance for themselves, their friends and family members. The social constructivist explanatory model indicates how people create their social world by imposing meaning to their practices, and view them as natural and inevitable – but only for the time being (as seen by the same NCC who were not circumcised) – since people tend to debunk, demystify and leave behind their beliefs quite spontaneously, without meaningful and logical continuation from one set of belief system to another (as seen by the NCC men who underwent medical MC). Hence the anecdotal example of one male circumcision in a NCC family leading to the circumcision being adopted by others in the family and friends may become examples of transitioning from one set of beliefs about MC to another set without any need for explanation. This phenomenon also highlights the role of *“perceived benefit”* construct of Health Belief Model which influences such a family-level acceptance.

Cost of medical circumcision was raised as an issue by the CC with a valid question on universal feasibility of the NCC opting for MC for the same reason. Cost could be a potential reason why CC might not want to change over from the practice of traditionally conducted circumcision to medically conducted circumcision. Affordability could be an important factor that could influence its acceptance and later on demand for MC in a resource limited country like India. Similar observations have been made in many other studies [30], [43], [44]. The community acceptance of programs such as family planning and institutional delivery is better accepted because they are incentivized in India [45]. An incentivized MC program for millions of males would be completely impractical and not feasible. It would add burden and cost to the health system which is already constrained. Undergoing any surgical procedure or paying for the procedure was not acceptable to the NCC as this was not their tradition. For similar reasons, the RHCPs in this study were not in favor of promoting male circumcision. However, certain countries have dealt with the cost issue by minimizing the cost of procedure and Indian health system could learn from them [46]. Using key messages in favor of voluntary medical male circumcision along with social interventions such as making peer groups of young men and married women as advocates could be some innovative interventions for reducing the barriers to voluntary medical male circumcision in India. It is possible to include adult MC services in the public health set ups and offer MC as one of the ‘options’ for HIV prevention especially targeted against high risk groups in India.

### Cultural disposition and the right informer

Communicating the medical arguments regarding benefits of MC and its potential role in preventing HIV acquisition is a socio-culturally sensitive issue. It is important to debate this issue in larger forums comprising of communities, theologians, religious leaders, clerics, doctors, program implementers and policy makers. Faith based HIV prevention programs have shown successes in African countries [47]. A recent study of interfaith theology of HIV/AIDS provides guidance on ‘spiritualization of condom’ by showing that it can save innocent lives [48]. Similar innovative messages and approaches might be required to ensure acceptance of adult MC in India.

While education and information play an influencing role in MC acceptance, this study also revealed that propagating ‘MC for HIV prevention’ may mislead the illiterate populations and it might lead to behavioral dis-inhibition and consequent risk of HIV acquisition. African studies have reported risky behaviors and multiple sexual partners among the circumcised men than the uncircumcised men [49], [50]. Appropriate communication strategy framework will have to be designed. Any information is grasped and internalized in accordance with one's own personal and cultural predispositions and it depends on information given to the informed and possessed by the informer. A fear of cognitive dissonance and incongruity surfaced when in spite of overall appreciation of MC among CC, skepticism and fear emerged among them and they were strongly averse to involving themselves in any promotional process for propagating MC among the NCC. According to Waldeck's (2002) norm theory [51], cultural predispositions have a powerful impact on the way any information is given. In case of a particularly sensitive issue of male circumcision in India, the informer for the NCC needs to be a doctor who can skillfully maneuver the filtration and incorporation of the information/evidence so that the information appears factual and nothing gets exaggerated or understated. Health care providers, community opinion leaders and NGO representatives along with theologians can come together to understand and explicate the doctrines and guide on better acceptance of MC by the communities.

The understanding of adult male circumcision does exist among NCCs but only in the context of ‘treatment for certain medical problems’. The challenge is how to understand the psychological mind-set of the NCC, who would logically be inclined to reject the medical argument owing to the historical denigration of “the circumcised”. The RHCPs experienced the need for repeatedly counseling the parents of children with medical problem/or adult patients about the exact benefits and side effects of MC and finally convincing them to undergo MC. Consultation and advice from the doctors is likely to carry greater value for better acceptability of MC among adults. This reveals the dynamics underlying the acceptability process of MC, which is driven not necessarily by paternalistic advice by the doctors, but through more effective communication and shared decision making between doctors and patients, based on evidences and benefits, an indication of the evolving doctor-patient relationship globally [52]. As raised by Hankins et al[53], it will take community conversations to create new social norms about male circumcision in previously non-circumcising communities. It might be important for women to speak in support of HIV prevention benefits and the desirability of male circumcision for their sexual partners and other male kin. It will require tailored communication strategies to create demand for adult MC services among the communities targeted, and it will take program planners to provide culturally matching supply of safe, acceptable, and accessible services.

Pain, as reported in a previous study [54], was one of the barriers for undergoing male circumcision. Some of the crucial contents for messages for better acceptability in India could be the assurance to provide easier options to undergo medical circumcision in the existing health systems and better follow-up and post-surgical pain management. The program can explore the usage of devices that negate the need for anesthetic injection (perceived to be painful) and sutures [55]. Messages on other benefits of MC including improved genital hygiene, reduction in chances of urinary tract infections and other STIs and relief from phimosis should also be stressed.

The study had some limitations. Interviews were not necessarily conducted by the interviewers of the same gender initially but later on with reports of refusals from CC, sites ensured interviewer to be of same gender. It was a qualitative exploratory study in an area that has religious and cultural sensitivity in India and some of the sensitive words and phrases could not be reported in this manuscript. We experienced challenges while tracking the traditional circumcisers for follow-up interviews. Owing to the religious and communal sensitivities involved, to avoid bringing many people together to discuss such a volatile topic, we preferred not to conduct focus group discussions (FGDs) [56]. Purposive sampling strategy was used for selection of participants. Hence, the findings may not be completely generalized and would need to be validated through systematically conducted large scale quantitative studies. However, the study has provided important clues and information that can provide direction for policy makers, program managers and researchers in the area of MC as an HIV prevention option.

### Conclusion

In this study, religion associated beliefs pertaining to male circumcision emerged to be critically sensitive and they could have grave implications as majority of the population in the country is non-circumcising. Neonatal male circumcision is the traditional practice in the CCs and mainly associated with religion. On the other hand, medical male circumcision is accepted in the NCC following appropriate medical advice. This provides a window of opportunity for promoting adult male circumcision, of course with due consideration to appropriate sensitivities towards religion and pain mitigation as part of community education and information. Any mass level propagation of MC as an HIV prevention program would face major resistance from the religious sections of the NCC for reasons of communal identity. Support from healthcare providers was also not observed as skepticism regarding trial results conducted abroad prevailed among them and they could not disassociate their social values and religious leanings. Training to bring attitudinal change among health care providers is recommended. The need for generating local evidence to convince the healthcare providers and the community in general emerged. It may be possible to propagate MC in India by secularizing and by projecting it as a medical procedure. It is advisable to provide MC as a Government Health Systems provided service and option for the select high-risk groups initially for prevention of HIV transmission. Taking decisions on the need to repeat clinical trials of circumcision efficacy in the country to produce local evidence and understanding the available MC trial results and their implications among the circumcising and non-circumcising communities in India need to be foremost in the minds of policy makers, program implementers and researchers.
